# In Situ Vapor Polymerization of Poly(3,4-ethylenedioxythiophene) Coated SnO_2_-Fe_2_O_3_ Continuous Electrospun Nanotubes for Rapid Detection of Iodide Ions

**DOI:** 10.3390/ma11112084

**Published:** 2018-10-24

**Authors:** Xiuru Xu, Wei Wang, Bolun Sun, Xue Zhang, Rui Zhao, Ce Wang

**Affiliations:** 1 Alan G. MacDiarmid Institute, Jilin University, Changchun 130012, China; bolun-sun@foxmail.com (B.S.); zhaoruikevin@163.com (R.Z.); 2 School of Advanced Materials, Peking University Shenzhen Graduate School, Shenzhen 518055, China; 3 State Key Laboratory of Urban Water Resource and Environment (SKLU-WRE), School of Municipal and Environmental Engineering, Harbin Institute of Technology, Harbin 150090, China; wangweirs@hit.edu.cn; 4 College of Resources and Environment, Jilin Agriculture University, Changchun 130118, China; zxue1987@163.com

**Keywords:** in situ vapor polymerization, electrospun nanotubes, ion-selective electrodes, iodide ion detection

## Abstract

In this work poly(3,4-ethylenedioxythiophene) (PEDOT) coated SnO_2_-Fe_2_O_3_ continuous nanotubes with a uniform core–shell structure have been demonstrated for rapid sensitive detection of iodide ions. The SnO_2_-Fe_2_O_3_ nanotubes were firstly fabricated via an electrospinning technique and following calcination process. An in situ polymerization approach was then performed to coat a uniform PEDOT shell on the surface of as-prepared SnO_2_-Fe_2_O_3_ nanotubes by vapor phase polymerization, using Fe_2_O_3_ on the surface of nanotubes as an oxidant in an acidic condition. The resultant PEDOT@SnO_2_-Fe_2_O_3_ core-shell nanotubes exhibit a fast response time (~4 s) toward iodide ion detection and a linear current response ranging from 10 to 100 μM, with a detection limit of 1.5 μM and sensitivity of 70 μA/mM/cm^2^. The facile fabrication process and high sensing performance of this study can promote a wide range of potential applications in human health monitoring and biosensing systems.

## 1. Introduction

Iodine is an important trace mineral and nutrient for humans, which is greatly needed for producing thyroid hormones in our body. Iodine deficiency is an important public health issue because it is a preventable cause of intellectual disability [1,2]. Therefore, rapid and sensitive detection of iodide ions is necessary for both diagnostic and pathological research. To date, a large variety of methods have been developed to detect iodide ions, including inductively coupled plasma mass spectrometry method (ICP-MS) [3], atomic absorption spectrometry method (AAS) [4], field-effect transition sensor (FET) [5], flow injection analysis (FIA) [6], etc. Among the developed approaches, voltammetric methods have gained great attention owing to their rapid, accurate, and high responses. The voltammetric method is strongly dependent on modification of traditional electrodes.

In the past few years, poly(3,4-ethylenedioxythiophene (PEDOT) has been reported to be utilized as a very stable and promising electrode material for iodide ion detection [7,8,9]. On the other hand, one-dimensional (1D)-based electrode modification nanomaterials for electrocatalytic oxidation such as nanotubes, nanorods, and nanofibers, have attracted considerable attention. Benefiting from their geometric advantages (light weight, high surface-to-volume ratio etc.), they can access aimed ions easily, provide excellent electron transport, efficient responses, and have sensitive properties [10,11,12,13]. However, fabrication of PEDOT composites with a 1D nanostructure is mainly realized by electrochemical polymerization with nanowire templates, such as ZnO nanowire arrays from a chemical vapor deposition or hydrothermal process [14,15,16], or with porous alumina [17] and a porous PC membrane [18]. Therefore, it is highly desirable to fabricate a 1D nanostructure PEDOT composite with a simple, low cost, and well-controlled approach.

Herein, we present the fabrication of continuous core-shell structured nanotubes composed of conducting polymers and metal oxides, which can be used as a good electrochemical iodide ion sensor. PEDOT coated SnO_2_-Fe_2_O_3_ core-shell nanotubes were prepared by combination of the electrospinning technique, calcination procedure, and an in situ vapor phase polymerization. In application to the determination of iodide ions, PEDOT coated SnO_2_-Fe_2_O_3_ core-shell nanotubes with modified glass carbon electrodes (GCE) exhibited high sensitivity with a low detection limit.

## 2. Results and Discussion

**Figure 1:**
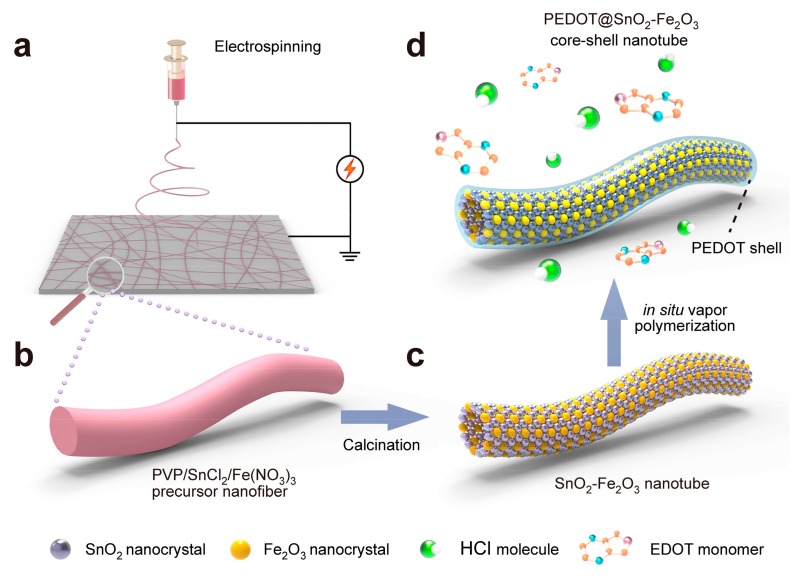
(**a**) Illustration of the electrospinning process used to fabricate PVP/SnCl_2_/Fe(NO_3_)_3_ precursor nanofibers. (**b**–**d**) Illustration of the in situ vapor polymerization method used to provide PEDOT@SnO_2_-Fe_2_O_3_ core-shell electrospun nanotubes by sequential calcination and exposure to HCl and 3,4-ethoxylene-dioxy-thiophene (EDOT) vapor under 8.0 Torr and 60 °C.

The detailed fabrication process of PEDOT coated SnO_2_-Fe_2_O_3_ core-shell nanotubes is schematically shown in Figure 1. Firstly, PVP poly(vinyl pyrrolidone) (PVP)/SnCl_2_/Fe(NO_3_)_3_ precursor nanofibers were obtained by an electrospinning technique. Resulting nanofibers were then calcined at 600 °C in air for 5 h to remove PVP and convert precursors into stable metal oxide crystals. As a result, heterojuncted SnO_2_-Fe_2_O_3_ nanotubes with a reddish color were obtained. An in situ vapor polymerization method was followed to fabricate PEDOT coated SnO_2_-Fe_2_O_3_ core-shell nanotubes. As-prepared SnO_2_-Fe_2_O_3_ nanotubes were exposed to HCl vapor and 3,4-ethoxylene-dioxy-thiophene (EDOT) vapor under ambient conditions (8.0 Torr at 60 °C, where 1.0 Torr ≈ 133 Pa). During this process a small amount of Fe_2_O_3_ nanocrystals were dissolved into Fe^3+^, which is also the oxidizing agent for polymerization of EDOT. Therefore, a uniform shell of PEDOT was grown and gradually polymerized on the surface of resulting SnO_2_-Fe_2_O_3_ nanotubes (Figure 1d). Compared to the liquid phase in situ polymerization reported in previous work [19,20], in situ vapor phase polymerization [21,22] enables the fabrication of materials with improved ordering, stability, and controllability at the nanoscale.

**Figure 2:**
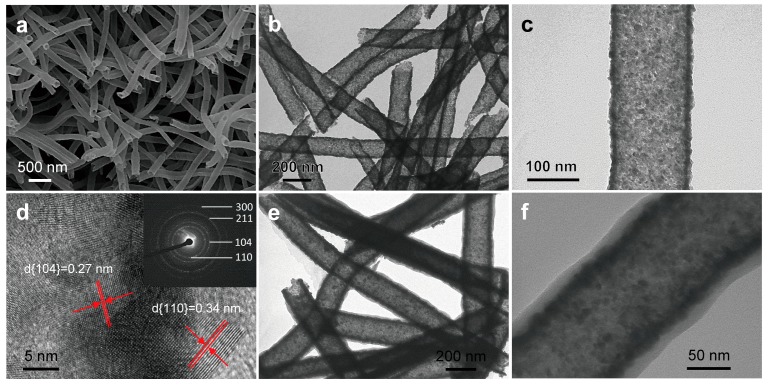
(**a**) SEM image, (**b**,**c**) TEM images, and (**d**) HRTEM image (Inset: SAED pattern) of SnO_2_-Fe_2_O_3_ heterojuncted electrospun nanotubes. (**e**,**f**) TEM images of PEDOT@SnO_2_-Fe_2_O_3_ core-shell electrospun nanotubes after in situ vapor polymerization. HRTEM image and SAED pattern could be indexed to SnO_2_ and α-Fe_2_O_3_. After in situ vapor polymerization, an approximately 10–20 nm-thick poly-3,4-ethylenedioxythiophene (PEDOT) shell was polymerized, resulting in the PEDOT-SnO_2_-Fe_2_O_3_ core-shell electrospun nanotube structure.

Morphologies of samples were characterized by SEM and TEM measurements. Figure 2a–c shows morphologies of as-prepared SnO_2_-Fe_2_O_3_ heterojuncted nanotubes. After calcination, Figure 2a–c clearly exhibit nanotube structures of as-prepared SnO_2_-Fe_2_O_3_ composed with small nanocrystals of SnO_2_ and Fe_2_O_3_, with a wall thickness of about 15–30 nm. The average diameter of SnO_2_-Fe_2_O_3_ nanotubes ranges from 150 to 200 nm and small nanocrystals from 5–15 nm. Figure 2d shows a high resolution transmission electron microscope (HRTEM) image of SnO_2_-Fe_2_O_3_ nanotubes, which exhibit the crystalline nature of the two metal oxides. Lattice fringe spacing of 0.34 nm is related to the (110) crystal plane of the rutile phase of SnO_2_ [23]. The other lattice fringe spacing of 0.27 nm is consistent with the (104) crystal plane of α-Fe_2_O_3_ [24]. From the selected area electron diffraction (SAED) pattern (inset of Figure 2d), the (110) and (211) crystal planes from the rutile phase of SnO_2_ and the (104) and (300) crystal planes from α-Fe_2_O_3_ are also found. Therefore, SnO_2_-Fe_2_O_3_ heterojuncted nanotubes were successfully synthesized in this work via a simple electrospinning technique combined with the calcination process. Formation of the nanotube structure of heterojuncted SnO_2_-Fe_2_O_3_ is likely due to phase separation among the precursors of tin (II) chloride, iron (III) nitrate, the PVP carrier, and the evaporation of solvents during the electrospinning process [25,26,27]. Subsequently, SnO_2_-Fe_2_O_3_ nanotubes were used for further preparation of PEDOT on their surface, through an in situ self-assembly vapor phase polymerization process. Morphology of the resulting PEDOT@SnO_2_-Fe_2_O_3_ core-shell nanotubes can be confirmed by TEM images in Figure 2e,f. The smooth, uniform PEDOT shell was polymerized on the surface of SnO_2_-Fe_2_O_3_ nanotubes with a sheath thickness of approximately 10–20 nm.

**Figure 3:**
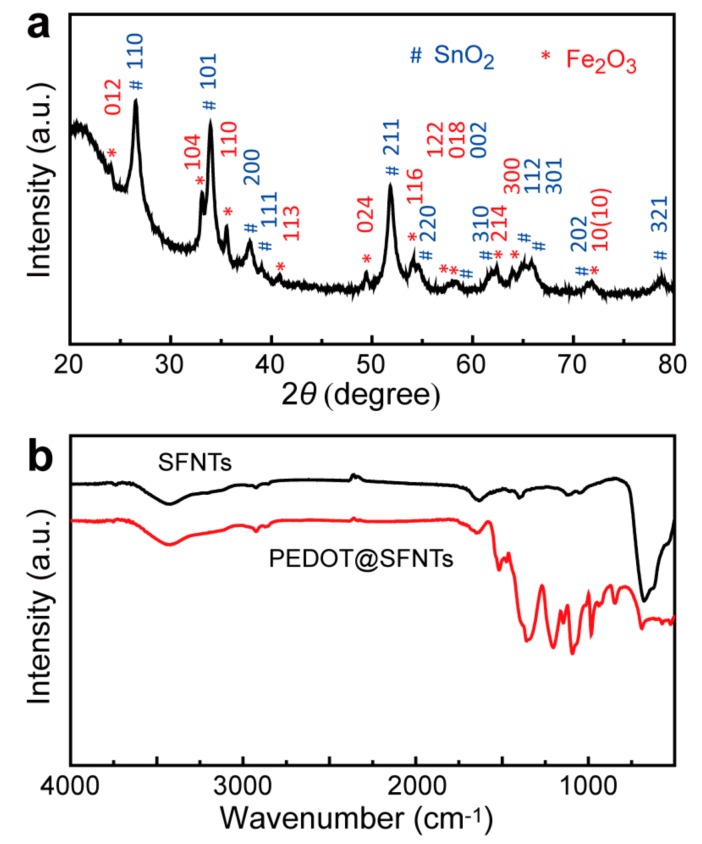
(**a**) X-ray diffraction of SnO_2_-Fe_2_O_3_ electrospun nanotubes (SFNTs); (**b**) The fourier transform infrared spectoscopy (FT-IR) spectra of SnO_2_-Fe_2_O_3_ nanotubes and PEDOT@SnO_2_-Fe_2_O_3_ core-shell nanotubes.

To further confirm the crystal structure of SnO_2_-Fe_2_O_3_ composite electrospun nanotubes, X-ray diffraction was characterized. As shown in Figure 3a, in agreement with the HRTEM and SAED results in Figure 2d, crystalline peaks at approximately 26.5°, 33.9°, 37.9°, 39.0°, 51.8°, 54.6°, 58.2°, 61.9°, 64.8°, 65.8°, 71.3° and 78.7° are ascribed to the (110), (101), (200), (111), (211), (220), (002), (310), (112), (301), (202) and (321) facet Bragg reflection of SnO_2_ respectively. All these diffraction peaks can be indexed as the tetragonal rutile structure of SnO_2_ (JCPDS 41-1445), further confirming the tetragonal rutile structure in samples [28,29]. Other diffraction peaks at approximately 24.1°, 33.1°, 35.6°, 40.8°, 49.4°, 54.0°, 57.5°, 57.9°, 62.4°, 64.0° and 71.8° are assigned to (012), (104), (110), (113), (024), (116), (112), (018), (214), (300) and (10(10)) of hematite (JCPDS 33-0664), respectively [30]. This indicates formation of the spinel α-Fe_2_O_3_ and successful fabrication of SnO_2_-Fe_2_O_3_ composite nanotubes in this work. After vapor polymerization the fourier transform infrared spectoscopy (FT-IR) spectrum of PEDOT@SnO_2_-Fe_2_O_3_ core-shell nanotubes shows sharp peaks at around 1517, 1474, 1358, and 1340 cm^−1^, which are ascribed to the stretching vibration of C=C and C–C in the thiophene ring. Bands at 1203 and 1092 cm^−1^ are assigned to the stretching vibration of the ethylenedioxy group (shown as Figure 3b) [12,31]. These results indicate formation of PEDOT on the surface of SnO_2_-Fe_2_O_3_ nanotubes.

**Figure 4:**
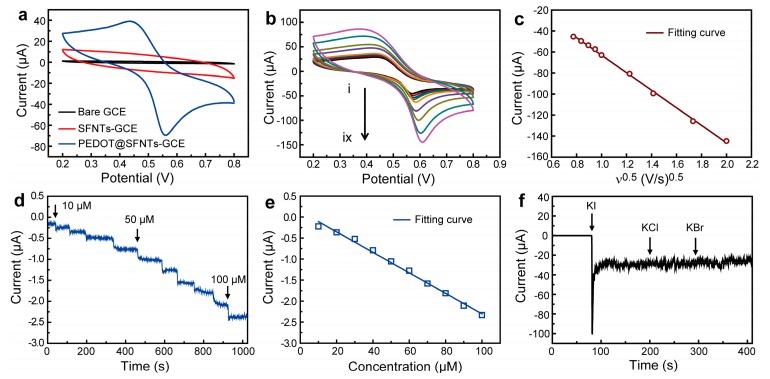
(**a**) Cyclic voltammogram (CV) curves of the bare glass carbon electrodes (GCE) (in black), SnO_2_-Fe_2_O_3_ electrospun nanotube modified GCE (SFNTs-GCE in red) and PEDOT@SnO_2_-Fe_2_O_3_ core-shell electrospun nanotube modified GCE (PEDOT@SFNTs-GCE in blue) in 1 mM KI and 0.1 M HClO_4_ with a scanning rate of 100 mV/s. (**b**) CV curves of as-prepared PEDOT-SnO_2_-Fe_2_O_3_ core-shell nanotube modified GCE in 1 mM KI and 0.1 M HClO_4_ solution at the scanning rate of (i) 60, (ii) 70, (iii) 80, (iv) 90, (v) 100, (vi) 150, (vii) 200, (viii) 300 and (ix) 400 mV/s. (**c**) Plot of the square root of scanning rate to peak current values. (**d**) Amperometric response of as-prepared PEDOT-SnO_2_-Fe_2_O_3_ core-shell nanotube modified GCE at 0.55 V to successive addition of KI into a 10 mL 0.1 M HClO_4_ solution with constant stirring in the KI range of 10–100 μM. (**e**) Calibration plot of current against various KI concentrations. (**f**) Interference behavior of as-prepared PEDOT@SnO_2_-Fe_2_O_3_ core-shell nanotube modified GCE illustrated by amperometric response at 0.55 V to successive addition of 0.5 mM KI, KCl, and KBr into 10 mL 0.1 M HClO_4_ solution stirred constantly, respectively.

Iodide ion sensing activities of as-prepared PEDOT@SnO_2_-Fe_2_O_3_ core-shell nanotubes were investigated. Figure 4a shows typical cyclic voltammogram (CV) curves of comparison between bare GCE, modified GCE with SnO_2_-Fe_2_O_3_ nanotubes, and modified GCE with PEDOT@SnO_2_-Fe_2_O_3_ core-shell nanotubes. It can obviously be noticed that no peaks can be observed from CV plots of bare GCE or modified GCE with SnO_2_-Fe_2_O_3_ nanotubes. Peak potentials of modified GCE with PEDOT@SnO_2_-Fe_2_O_3_ core-shell nanotubes were about 0.437 V and 0.558 V and peak currents were about 38.9 μA and −69.6 μA respectively. Response peaks with an obviously high current, from oxidation of iodide ions on GCE modified with PEDOT-SnO_2_-Fe_2_O_3_ core-shell nanotubes, indicated their high iodide ion sensitivity. Figure 4b,c shows the effect of scanning rate on electrochemical oxidation of iodide ions on GCE modified by PEDOT-SnO_2_-Fe_2_O_3_ core-shell nanotubes. It can be observed that the current response to iodide ions increases with increasing scanning rate (Figure 4b). Figure 4c shows the fitting plot of the response peak current to the square root of different scanning rates. Ranging from the scanning rate of 60 to 400 mV/s, the linear regression equation is: I (μA) = −83.45*v*^1/2^ − 20.479 (R^2^ = 0.9984). Therefore, the dynamic mechanism of detecting iodide ions on GCE modified by PEDOT@SnO_2_-Fe_2_O_3_ core-shell nanotubes is controlled by diffusion processes, according to the proportional relation between peak current and the square root of scanning rate [32].

The amperometric response of GCE modified with PEDOT@SnO_2_-Fe_2_O_3_ core–shell electrospun nanotubes is shown in Figure 4d. It was performed by successively adding KI into 10 mL 0.1 M HClO_4_ solution. It shows a rapid and sensitive response to the change of KI concentration with an excellent linear response within the range from 10 μM to 100 μM of iodide ions. Calculated sensitivity of 70.1 μA/mM/cm^2^ was obtained with a detection limit of 1.5 μM (based on S/N = 3) [33]. Average response time was approximately 4 s. The linearity regression equation is: I (μA) = −0.0244C + 0.136 (R^2^ = 0.9935) (Figure 4e). In addition, we also tested the anti-interference ability against other halide ions (Cl^−^ and Br^−^) on the modified GCE by as-prepared PEDOT-SnO_2_-Fe_2_O_3_ core-shell nanotubes (Figure 4f). It can be clearly observed that there is almost no current response to 0.5 mM concentration of Cl^−^ and Br^−^. Comparison of the detection of iodide ions by various methods is summarized in Table 1. Accordingly, results show that voltammetric methods by PEDOT-SnO_2_-Fe_2_O_3_ core-shell nanotube modified GCE have achieved a rapid response time of approximately 4 s, which is much faster than the response time of other measurements. The iodide ion detection mechanism may be due to formation of charge-transfer complexes with iodine molecules. Iodine is the product of iodide ion oxidation on modified GCE with PEDOT@SnO_2_-Fe_2_O_3_ core-shell nanotubes [7,34,35]. The conductive polymer PEDOT is the material which is mainly sensitive towards iodide ion detection, forming charge-transfer complexes with the product iodide, which acts as a donor and the latter acting as acceptors. Furthermore, good sensitivity and fast response time could be attributed to the 1D continuous electrospun core-shell nanotube structure of PEDOT@SnO_2_-Fe_2_O_3_. The core-shell nanotube structure can facilitate oxidation of iodide ions and formation of PEDOT-iodine charge-transfer complexes, which can react both at the inner wall and outer wall of PEDOT@SnO_2_-Fe_2_O_3_ nanotubes. It can also improve the rate of charge carriers in the PEDOT transversing along the nanotubes.

## 3. Materials and Methods

### 3.1. Preparation of PVP/SnCl_2_/Fe(NO_3_)_3_ Precursor Electrospun Nanofibers

All chemicals were purchased from Aladdin Industrial (Shanghai, China), unless mentioned otherwise and were used as received without any further purification. 4 wt% of tin (II) chloride dihydrate (SnCl_2_•2H_2_O), 2 wt% of iron (III) nitrate nonahydrate (Fe(NO_3_)_3_•9H_2_O) were dissolved in 42 wt% of ethanol (EtOH) firstly and then 42 wt% of *N*′,*N*′-dimethylformamide (DMF) was added under vigorous stirring. After 15 min, 10 wt% of poly(vinyl pyrrolidone) (PVP, *M*_W_ = 1,300,000, Sigma-Aldrich, Shanghai, China) was added with vigorous stirring for 30 min. Subsequently, precursor solution was loaded into a glass syringe with the tip inner diameter of approximately 1 mm. It was applied with a 15 kV DC voltage between tip and aluminum foil plate collector with a distance of 15 cm.

### 3.2. Preparation of PEDOT@SnO_2_-Fe_2_O_3_ Core-Shell Nanotubes

As-prepared PVP/SnCl_2_/Fe(NO_3_)_3_ precursor electrospun nanofibers were peeled off from the collector and placed in a crucible. As described in Figure 1, a 600 °C calcination process for 5 h was followed to remove PVP and obtain the heterojuncted SnO_2_-Fe_2_O_3_ electrospun nanotubes. Then, an in situ vapor phase polymerization was performed by exposing heterojuncted SnO_2_-Fe_2_O_3_ electrospun nanotubes to HCl vapor and monomer 3,4-ethoxylene-dioxy-thiophene (EDOT) vapor under ambient conditions. After in situ vapor phase polymerization for 6–12 h, a uniform shell of PEDOT was grown and gradually polymerized on the surface of resulting SnO_2_-Fe_2_O_3_ nanotubes and the PEDOT@SnO_2_-Fe_2_O_3_ core-shell nanotubes were obtained.

### 3.3. Fabrication of PEDOT@SnO_2_-Fe_2_O_3_ Core-Shell Nanotube Modified GCE

As-prepared PEDOT-SnO_2_-Fe_2_O_3_ core-shell nanotubes were washed with deionized (DI) water and ethanol for several times. Then, PEDOT@SnO_2_-Fe_2_O_3_ nanotubes were dispersed into ethanol to prepare the dispersion, the concentration was 5 mg/mL. Subsequently, 10 μL of as-prepared PEDOT@SnO_2_-Fe_2_O_3_ nanotube/ethanol dispersion was dropped onto a clean GCE surface and then evaporated in air at room temperature. Therefore, the PEDOT@SnO_2_-Fe_2_O_3_ core-shell nanotube modified GCE was obtained and used as the working electrode. All solutions were purged with N_2_ for 15 min to remove O_2_ before using and all experiments were performed in the glove box under a N_2_ atmosphere [9]. Electrochemical performance of the PEDOT@SnO_2_-Fe_2_O_3_ core-shell nanotube modified GCE was investigated by using CV scanning with a CHI660B Electrochemical Station (Shanghai CHENHUA instrument Co., Ltd., Shanghai, China). In a three-electrode system, the PEDOT-SnO_2_-Fe_2_O_3_ core-shell nanotube modified GCE was used as the working electrode. A platinum wire and saturated calomel electrode (SCE) were used as the counter electrode and reference electrode respectively.

## 4. Conclusions

In summary, PEDOT@SnO_2_-Fe_2_O_3_ core-shell nanotubes with well-defined morphology have been successfully fabricated via an electrospinning technique, calcination procedure, and in situ vapor polymerization process. As-prepared PEDOT@SnO_2_-Fe_2_O_3_ core-shell nanotubes exhibited high current response and rapid response time toward detection of iodide ions. It is anticipated that this electrode could be used for construction of high-performance biosensors.

## Figures and Tables

**Table 1:** Brief summary of measurements reported on iodide ion detection.

Detecting Method	LOD	Linear Range	Linear R^2^	Response Time
ICP-MS [6] ^a^	2.5 μg/L	25–355 μg/L		5 min
AAS [7] ^b^	2.75 μg/L	11–350 μg/L	0.998	~3 min
FET [8] ^c^	0.03 μM	0.1 μM–10 mM		>100 s
FIA [9] ^d^	500 μg/L	1–10 mg/L	0.999	2 min
Current work	1.5 μM	10–100 μM	0.9935	~4 s

^a^ ICP-MS is for inductively coupled plasma mass spectrometry method; ^b^ AAS is for atomic absorption spectrometry method; ^c^ FET is for field-effect transition sensor; ^d^ FIA is for flow injection analysis.
